# The non-steroidal mineralocorticoid receptor blocker esaxerenone reduces glomerular hyperfiltration and albuminuria

**DOI:** 10.1093/ndt/gfaf232

**Published:** 2026-05-29

**Authors:** Rie Tatsugawa, Kengo Kidokoro, Tsukasa Iwakura, Akira Hirano, Eriko Kajimoto, Masanobu Takasu, Masafumi Wada, Hiroshi Kitao, Yoshihisa Wada, Georgina Gyarmati, Seiji Kishi, Hajime Nagasu, David Z. I. Cherney, Janos Peti-peterdi, Tamaki Sasaki, Naoki Kashihara

**Affiliations:** 1 Department of Nephrology and Hypertension, Kawasaki Medical School, Kurashiki, Okayama, Japan; 2 Department of Physiology and Neuroscience and Department of Medicine, Zilkha Neurogenetic Institute, University of Southern California, Los Angeles, CA, USA; 3 Division of Nephrology, Department of Medicine, University Health Network, University of Toronto, Toronto, Ontario, Canada; 4 Kawasaki Medical School, Kawasaki Geriatric Medical Center, Kurashiki, Okayama, Japan

**Keywords:** adenosine, diabetic kidney disease, glomerular haemodynamics, non-steroidal mineralocorticoid receptor antagonist, tubuloglomerular feedback

## Abstract

**Background.:**

Diabetic kidney disease (DKD) is a major cause of chronic kidney disease, with glomerular hyperfiltration contributing to its progression. Esaxerenone, a non-steroidal mineralocorticoid receptor antagonist (MRA), reduces albuminuria, but its precise mechanism remains unclear. Mineralocorticoid receptor (MR) activation is implicated in tubuloglomerular feedback (TGF) dysregulation, and we hypothesized that MR inhibition attenuates albuminuria by mitigating glomerular hyperfiltration.

**Methods.:**

To investigate the effects of esaxerenone, we used aldosterone-induced MR activation rats and type 2 diabetic (db/db) mice. *In vivo* multiphoton imaging was performed to assess the single-nephron glomerular filtration rate (SNGFR) and arteriolar diameters. Macula densa (MD) cells were used to examine MR activation’s impact on TGF.

**Results.:**

In aldosterone-infused rats, MR activation induced glomerular hyperfiltration via afferent arteriolar dilation, which was attenuated by esaxerenone. In db/db mice, esaxerenone reduced SNGFR and urinary albumin excretion while increasing urinary adenosine levels, effects reversed by A1aR blockade. In MD cells, MR activation increased nitric oxide (NO) production and reduced Na^+^ -K^+^ -2Cl^−^ cotransporter membrane expression, both of which were mitigated by MRA or neuronal nitric oxide synthase inhibition.

**Conclusion.:**

These findings suggest that esaxerenone restores TGF function via adenosine signalling, attenuating glomerular hyperfiltration and reducing albuminuria. This study provides novel insights into the albuminuria-lowering effects of MR blockade in DKD and supports the therapeutic potential of esaxerenone.

## GRAPHICAL ABSTRACT

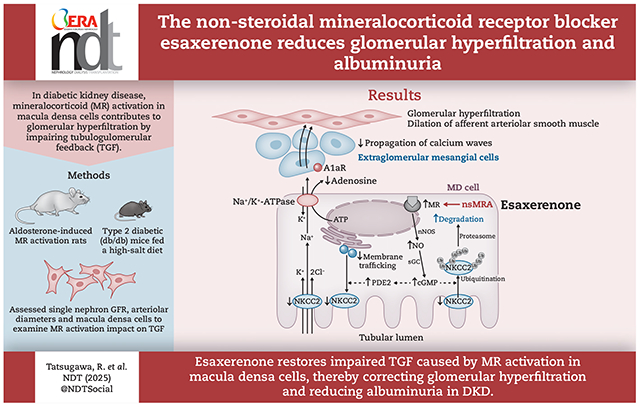

## INTRODUCTION

According to the 2023 International Society of Nephrology Global Kidney Health Atlas, there are 850 million individuals worldwide affected by chronic kidney disease (CKD) [1]. Diabetic kidney disease (DKD) represents one of the leading causes of CKD. A comprehensive understanding of its pathophysiology and the development of effective therapeutic approaches are acknowledged as critical and urgent issues. Finerenone, a non-steroidal mineralocorticoid receptor antagonist (MRA), has demonstrated renal protective effects in patients with CKD and type 2 diabetes in the FIDELIO-DKD (NCT02540993) trial [2].

MRAs, with a steroidal structure, are associated with a high risk of hyperkalaemia in cases of renal impairment, limiting their clinical application in CKD patients. Consequently, the mechanisms through which MRAs slow the progression of CKD remain largely unexplored. Animal studies have shown that combination therapy with esaxerenone and olmesartan reduces proteinuria independent of systolic blood pressure (BP) [3]. The improvement in proteinuria by MRAs has been linked to mechanisms like podocyte protection and reduced inflammation [4, 5]. Recent clinical trials of non-steroidal MRAs (nsMRAs) have shown an initial decline in estimated glomerular filtration rate (eGFR) after administration, followed by long-term preservation of renal function compared with placebo. Furthermore, an increase in eGFR and urinary albumin excretion was observed after discontinuation of the MRA [6]. This pattern of eGFR is similar to that observed in clinical trials of sodium–glucose co-transporter 2 inhibitors, suggesting that suppression of MR activity has the potential to correct glomerular hyperfiltration. Thus MR activity likely plays a functional role in regulating GFR and could be a significant factor in the renoprotective effects of nsMRAs in DKD.

Recent findings suggest that MR activation regulates glomerular haemodynamics via the tubuloglomerular feedback (TGF) mechanism [7]. MR activation induced by aldosterone stimulation in MMDD1 cells leads to TGF attenuation through increased nitric oxide (NO) production. However, few studies have investigated the involvement of MR activation in the glomerular hyperfiltration observed in diabetic nephropathy and the inhibitory mechanisms of MRAs.

We hypothesize that MR activation in macula densa (MD) cells contributes to glomerular hyperfiltration by impairing TGF and that the nsMRA esaxerenone attenuates albuminuria in DKD by restoring TGF function and reducing hyperfiltration.

## MATERIALS AND METHODS

### Animals

The experimental protocols followed in the current study were approved by the Ethics Review Committee for Animal Experimentation at Kawasaki Medical School, Kurashiki, Japan (21-088, 24-066). Male Sprague Dawley (SD) rats ( *n* = 3–4) were purchased from CLEA Japan (Tokyo, Japan). Male homozygous Lepr^db/db^ mice were diabetic and heterozygous Lepr^db/m+^ mice were used as controls (denoted as db/db and db/m^+^, respectively). db/db and db/m^+^ mice were purchased from CLEA Japan. A total of 58 animals were used in this study. All animals were maintained under controlled temperature and humidity conditions with 12 h light/dark cycles. SD rats were fed standard laboratory animal chow and had free access to tap water. db/m^+^ mice and db/db mice were fed a high salt diet [3% sodium chloride (NaCl)] and had free access to tap water.

### Protocols

Male SD rats (7–8 weeks) were assigned to one of the groups as follows: control ( *n* = 4), Aldo infusion (1.0 *μ*g/h; *n* = 4) or Aldo + esaxerenone (Esax; 3 mg/kg/day; *n* = 4). Aldosterone was obtained from Sigma-Aldrich (St. Louis, MO, USA). Esaxerenone was provided by Daiichi Sankyo (Tokyo, Japan). Osmotic minipumps (Durect, Cupertino, CA, USA) were implanted subcutaneously to infuse either the saline (control) or Aldo. Esax was administered orally once daily. After 3 days of drug administration, BP, heart rate and body weight were measured, followed by *in vivo* imaging of the rats. After *in vivo* imaging, the right kidney was collected and a segment of it was used for histologic examination. The BP and pulse rate were measured using the tail-cuff method with an automatic sphygmomanometer (BP98A; Softron, Tokyo, Japan).

Male db/m^+^ and db/db mice (9–10 weeks) were fed a high-salt (3% NaCl) diet for 8 weeks. db/db mice were treated with Esax (3 mg/kg/day) orally for 8 weeks by gavage and the following three groups were established: db/m^+^ group, db/db group and db/db + Esax group. The number of mice used was as follows: 6 mice per group for urine, serum and tissue collection; 3–5 mice per group for single-nephron GFR (SNGFR) measurement and 6–10 mice per group for renal haemodynamics assessment, including those from which data could not be obtained by *in vivo* imaging. Serum glucose, potassium and creatinine were measured at a central clinical laboratory (SRL, Tokyo, Japan). Urinary albumin levels were determined by enzyme-linked immunosorbent assay (ELISA) using a murine microalbuminuria ELISA kit (Albuwell M; Ethos Biosciences, Philadelphia, PA, USA). Urinary creatinine was measured using commercially available lab assay creatinine (FUJIFILM Wako Pure Chemical, Osaka, Japan). The urinary adenosine was determined using fluorometric adenosine assay (BioVision, Milpitas, CA, USA).

Ten-week-old male db/db mice were fed a high-salt (3% NaCl) diet for 4 weeks. db/db mice were treated with Esax (3 mg/kg/day) orally and an adenosine A1 receptor (A1aR) antagonist (8-cyclopentyl-1,3-dipropylxanthine; Abcam, Cambridge, UK) via intraperitoneal injection for 4 weeks and the following three groups were established: db/db group, db/db + Esax group and db/db + Esax + A1aR antagonist group.

### Multiphoton *in vivo* imaging

*In vivo* imaging was performed as previously described [8]. The images were captured using a Nikon A1R-MP multiphoton confocal microscope equipped with an inverted imaging system and an Apo LWD 25 × 1.10 W DIC N2 objective lens. Bovine serum albumin, Alexa Fluor 594 conjugate (A13101) and lucifer yellow (LY; L453) were purchased from Molecular Probes (Eugene, OR, USA). Bovine serum albumin, Alexa Fluor 594 conjugate (0.3 ml for rats or 0.1 ml for mice at a 5 mg/ml concentration, administered through the carotid artery catheter; Invitrogen, Waltham, MA, USA) was used for labelling the plasma flow, whereas LY (50–100 *μ*l for rats or 10–20 *μ*l for mice at a 10 mg/ml concentration, administered through the carotid artery catheter by bolus injection; Invitrogen) was used for measuring SNGFR. Multiphoton imaging was performed using an excitation wavelength of 800 nm and Alexa Fluor 594 conjugate and LY were detected through 595/50- and 525/50-nm bandpass filters, respectively.

### Measurement of SNGFR and glomerular arteriolar diameter

SNGFR was measured as previously described [8]. After the left kidney was exteriorized, a cortical slice < 1 mm thick was removed and used to image the microcirculation of the most superficial glomeruli for the observation of the glomerular arterioles. The glomeruli used for observation were limited to those with intact nephrons, including the Bowman’s capsule, glomerulus and proximal tubules.

### Histologic assessment

Sections (4-*μ*m thick) were prepared from renal tissue samples embedded in paraffin and stained with periodic acid–Schiff (PAS) and Masson’s trichrome. Morphologic changes in the glomerular capillary wall were examined by scanning and transmission electron microscopy.

### Cell culture

The MD cells (MDgeo cell line) used in this study have been described previously [9]. MD cells were cultured at 33°C in the presence of NGF (0.1 *μ*g/mL) for proliferation. Differentiation was induced by adding nerve growth factor (NGF; 0.1 *μ*g/ml) and incubating the cells at 37°C for 10–14 days. The stimulation with aldosterone (100 nM) was performed for 24 hours, while esaxerenone (1 *μ*M) and the neuronal nitric oxide synthase inhibitor (nNOSi) N-(4S)-(4-amino-5- [aminoethyl]aminopentyl)-N′-nitroguanidine tris(trifluoroacetate) salt (sc-215427; Santa Cruz Biotechnology, Dallas, TX, USA) were administered 3 hours prior to aldosterone addition. During the experiments, MDgeo cells were maintained at 37°C under conditions where an orbital shaker was used to generate a gentle flow in the culture medium. In some experiments, spironolactone (100 nM; FUJIFILM Wako Pure Chemical) was used.

### Western blotting

Cell lysates were extracted with extraction buffer or sample buffer, as described previously [10]. Protein samples were subjected to immunoblotting analysis with the anti-NKCC antibody (clone T4, Developmental Studies Hybridoma Bank, University of Iowa, Iowa City, IA, USA), cyclooxygenase-2 (COX-2; ab15191, Abcam), MR (rMR365-4D6, Developmental Studies Hybridoma Bank, University of Iowa) and serum- and glucocorticoid-regulated kinase 1 (SGK1; ab32374, Abcam). The T4 antibody detects both NKCC1 and NKCC2. However, since NKCC1 expression is low in the kidneys, its specificity was not considered in this study. For surface protein analysis, plasma membrane proteins were isolated from MD cell preparations using a commercial membrane protein extraction kit (A44393, Thermo Fisher Scientific, Waltham, MA, USA) according to the manufacturer’s instructions.

### Detection of intracellular NO levels

DAX-J2 Red (Cosmo Bio, Tokyo, Japan; Ex/Em = 588/610 nm) was used for the visualization of intracellular NO. Quantification was performed based on the fluorescence intensity per cell area, with 15–20 cells randomly selected from each group. A Cell Meter Fluorimetric Intracellular Nitric Oxide Assay Kit (AAT Bioquest, Sunnyvale, CA, USA; #16359) was used to detect intracellular NO levels in MDgeo cells according to the manufacturer’s protocol. Fluorescence increase was measured using a microplate reader at Ex/Em = 650/680 nm.

### Immunofluorescence staining

To label NKCC2 on MDgeo cells, the T4 antibody was conjugated with Alexa Fluor 488 using the Lightning-Link Rapid Conjugation System (Innova Biosciences, Cambridge, UK). Frozen kidney sections (5-*μ*m thick) were prepared using a cryostat and mounted on glass slides. After air drying, the sections were blocked with 5% donkey serum for 1 hour at room temperature and then incubated overnight at 4°C with a rabbit polyclonal antibody against NKCC2 (SLC12A1; Proteintech, Rosemont, IL, USA; #18970-1-AP). After washing, the sections were incubated for 1 hour at room temperature with a fluorescein isothiocyanate–conjugated swine anti-rabbit immunoglobulin G secondary antibody (Dako/Agilent, Santa Clara, CA, USA; #F0205).

### Statistical analyses

Statistical analyses were performed using GraphPad Prism 7 software (GraphPad Software, Boston, MA, USA). Comparisons between two groups were performed using an unpaired or paired two-tailed Student’s *t*-test or Mann-Whitney U test. Comparisons between multiple groups with normal distributions were performed using one-way analysis of variance followed by the Tukey’s multiple comparison test, whereas the Kruskal–Wallis test followed by Dunn’s multiple comparison test was used for groups with non-normal distribution. All values are expressed as the mean ± standard error of the mean (SEM). Statistical significance was set at *P* < .05.

## RESULTS

### Physiological and biochemical data and glomerular haemodynamics in MR activation induced by aldosterone

**Figure 1:**
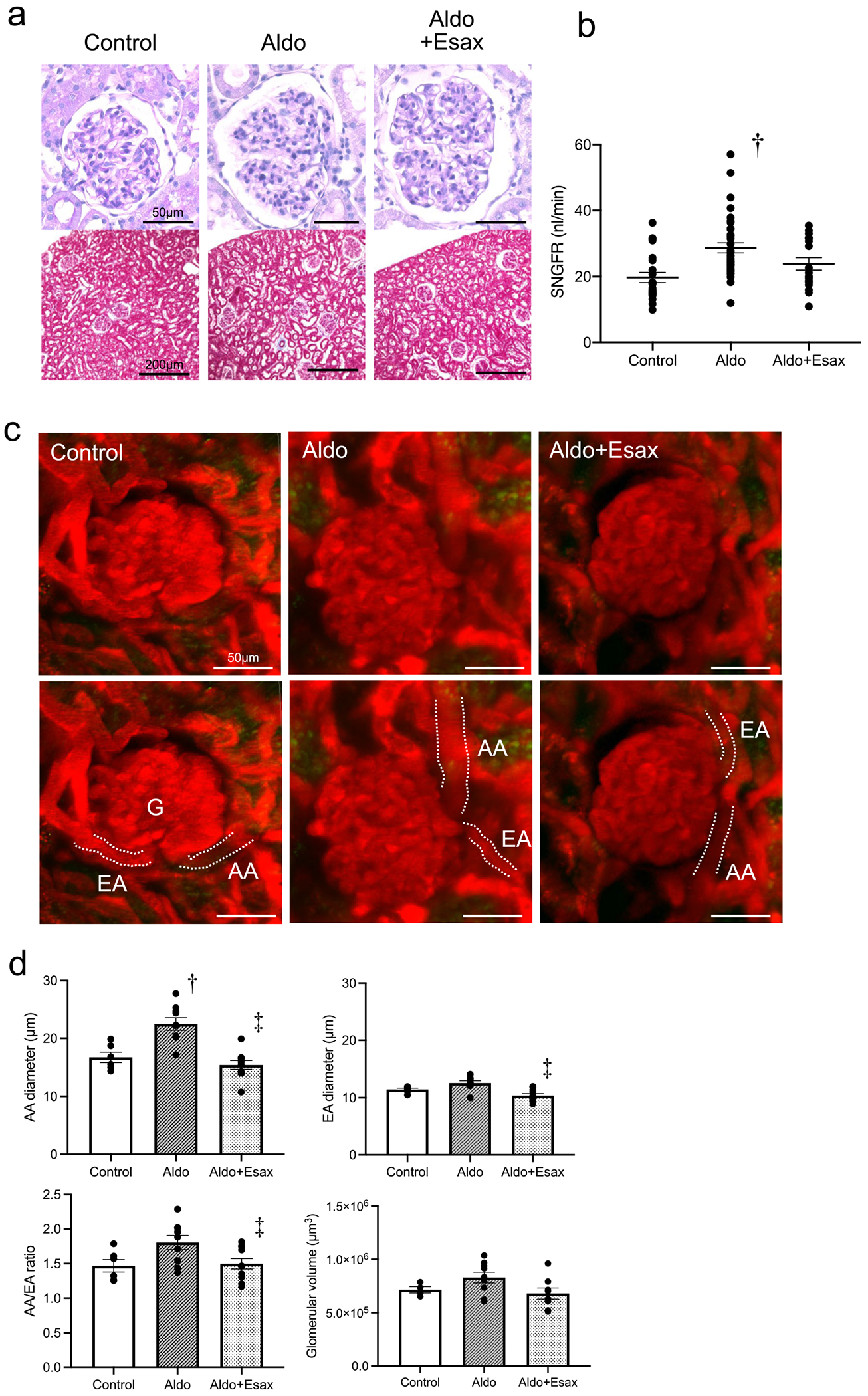
Histological findings and glomerular haemodynamic changes after MR activation by aldosterone administration in SD rats. **(a)** Representative images of glomeruli stained with PAS (upper panels; scale bar = 50 *μ*m) and Masson’s trichrome (lower panels; scale bar = 200 *μ*m). **(b)** SNGFR. **(c)** Intravital multiphoton microscopy images of AA and EA. Scale bar = 50 *μ*m. **(d)** Quantification of AA and EA diameters, AA:EA ratio and glomerular volume. Data are presented as mean ± SEM. G: glomerulus. ^†^ *P* < .05 versus control; ^‡^ *P* < .05 versus aldosterone.

To assess alterations in renal haemodynamics induced by MR activation, we administered aldosterone to SD rats and analysed the resulting changes. In the aldosterone-treated group, BP significantly increased compared with the control group; however, this elevation was markedly mitigated by administration of esaxerenone (Table 1). Histological analysis using PAS and Masson’s trichrome staining demonstrated no significant changes in glomerular injury or interstitial fibrosis among the three groups (Fig. 1a). *In vivo* imaging analysis demonstrated a significant increase in SNGFR in the aldosterone-treated group compared with the control group, indicating glomerular hyperfiltration (Fig. 1b). Additionally, the aldosterone-treated group exhibited afferent arteriolar dilation compared with the control group, which was corrected by treatment of esaxerenone. The diameter of efferent arterioles was significantly reduced in the esaxerenone group compared with the aldosterone-treated group. The afferent:efferent arteriole ratio (AA:EA) showed a trend toward an increase in the aldosterone-treated group compared with the control group, but this difference was not statistically significant. In contrast, esaxerenone significantly reduced the ratio compared with the aldosterone group. Glomerular size remained unaffected by any treatment (Fig. 1c, d). These findings indicate that MR activation induced by aldosterone administration leads to glomerular hyperfiltration via afferent arteriole dilation.

### Glomerular haemodynamics and correction of glomerular hyperfiltration by esaxerenone in a diabetic nephropathy model mouse

**Figure 2:**
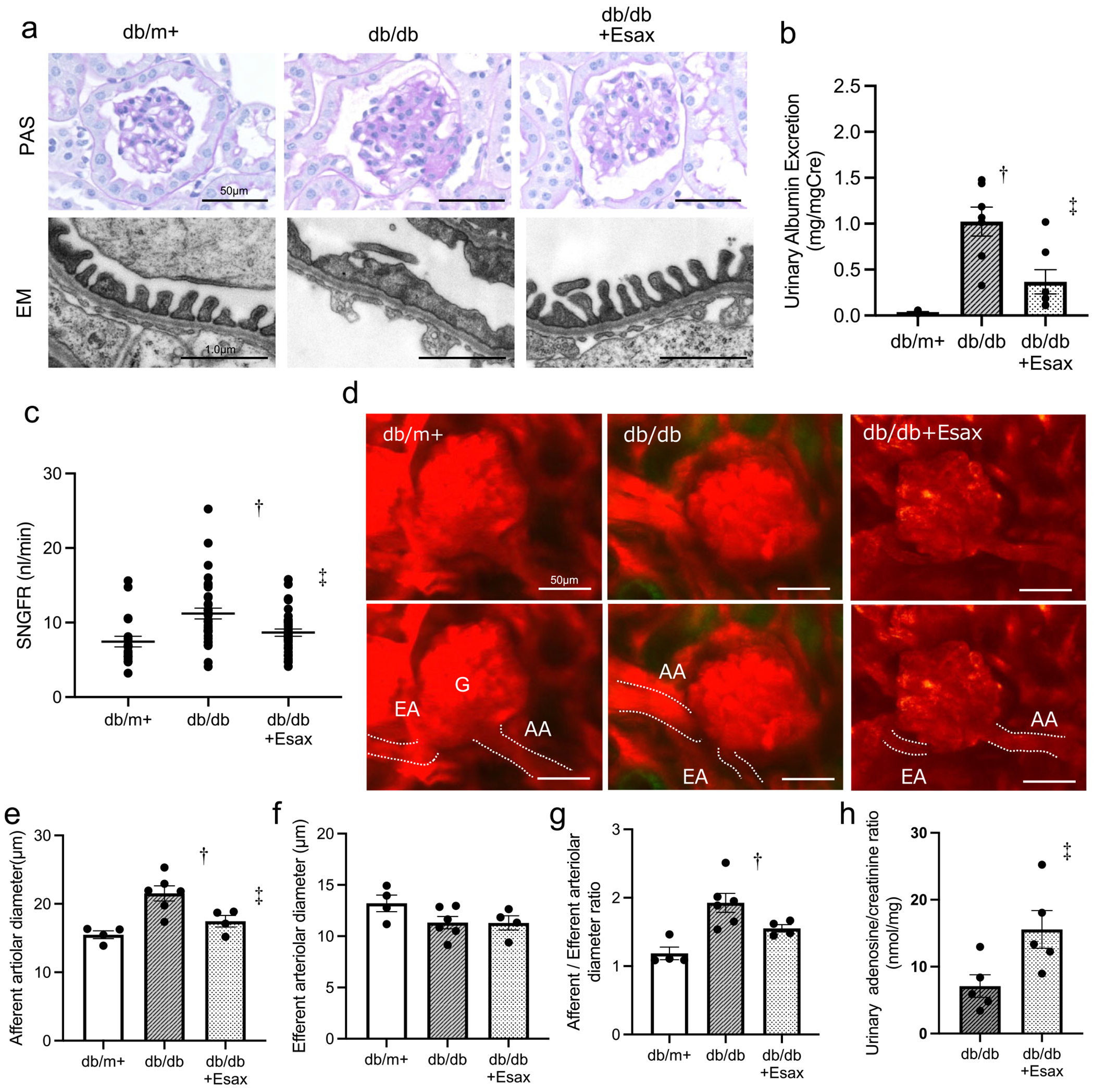
Histological findings and glomerular haemodynamics in db/db mice treated with esaxerenone. **(a)** Representative images of PAS staining (upper panels; scale bar = 50 *μ*m) and ultrastructural changes observed by electron microscopy (lower panels; scale bar = 1.0 *μ*m). **(b)** Urinary albumin excretion. **(c)** SNGFR. **(d)** Intravital multiphoton microscopy images showing AA and EA. Scale bar = 50 *μ*m. **(e)** Afferent arteriole diameter. **(f)** Efferent arteriole diameter. **(g)** Ratio of afferent to efferent arteriole diameter (AA/EA ratio). **(h)** Urinary adenosine/creatinine ratio. G: glomerulus. Data are presented as mean ± SEM. ^†^ *P* < .05 versus db/m^+^; ^‡^ *P* < .05 versus db/db.

The effects of esaxerenone on renal haemodynamics were investigated using type 2 diabetes model mice. In the db/db and db/db + Esax groups, significant reductions in systolic BP, pulse rate and serum creatinine level were observed compared with the db/m^+^ group, while body weight and blood glucose levels were significantly increased. However, no significant differences were noted between the db/db and db/db + Esax groups (Table 2). It should be noted that despite a high-salt diet, none of the groups exhibited a significant increase in BP (see Supplementary Fig. S1). Previous studies demonstrating salt-induced hypertension in db/db mice used higher salt concentrations (4–8% NaCl) and/or older animals (≈34 weeks) [11, 12], whereas our protocol employed a milder salt content and younger mice (8–15 weeks), which were likely insufficient to elevate BP. In the db/db mice, mesangial matrix expansion and morphological changes in podocytes, such as foot process effacement, were observed. These pathological changes were ameliorated by esaxerenone treatment (Fig. 2a). Detailed renal morphological findings are provided in the Supplementary Data (see Supplementary Fig. S2). Fibrotic changes were scarcely observed in db/db mice and no clear antifibrotic effect of esaxerenone was identified. Urinary albumin excretion was significantly higher in the db/db group compared with the db/m^+^ group but was reduced by esaxerenone treatment (Fig. 2b). Additionally, an increase in SNGFR was observed in the db/db group, which was attenuated by esaxerenone treatment (Fig. 2c). Afferent arteriolar dilation was observed in the db/db mice compared with the db/m^+^ group and this dilation was corrected by esaxerenone treatment. No significant change was detected in the diameter of the efferent arteriole in each group (Fig. 2d–f). The AA:EA ratio was elevated exclusively in the db/db group (Fig. 2g). Moreover, urinary adenosine levels were significantly increased in the db/db + Esax group compared with the db/db group (Fig. 2h). These findings suggest that the corrective effect of MR blockade on glomerular hyperfiltration in DKD is mediated through adenosine signalling via TGF.

### Attenuation of the MR blocker–induced correction of glomerular hyperfiltration in diabetic nephropathy by an adenosine receptor antagonist

**Figure 3:**
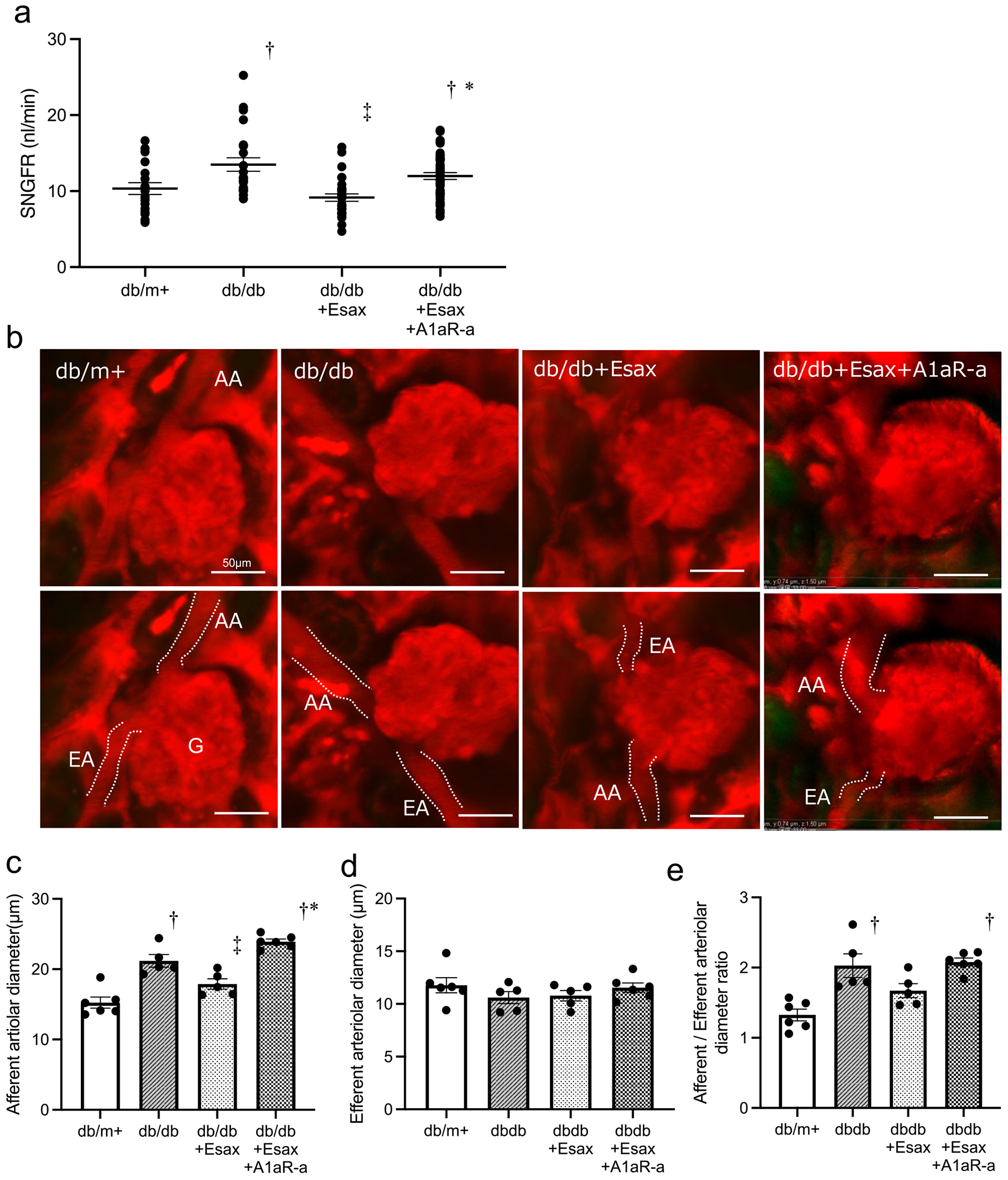
Glomerular haemodynamic changes in db/db mice treated with esaxerenone and an A1aR antagonist. **(a)** SNGFR. **(b)** Intravital multiphoton microscopy images of AA and EA. Scale bar = 50 *μ*m. **(c, d)** Quantification of AA diameter and EA diameter. **(e)** Ratio of AA to EA diameter. G: glomerulus. Data are presented as mean ± SEM. ^†^ *P* < .05 versus db/m^+^. ^‡^ *P* < .05 versus db/db. * *P* < .05 versus db/db + Esax.

To clarify the relationship between MR activity and adenosine signalling via TGF, an additional group treated with an A1aR antagonist was included in the analysis. Similar to the previous experiments, the db/db group exhibited a significant increase in SNGFR compared with the db/m + group, indicating glomerular hyperfiltration. In the db/db + Esax group, SNGFR was significantly reduced compared with the db/db group, demonstrating the corrective effect of esaxerenone on glomerular hyperfiltration. However, in the A1aR-a co-treatment group, SNGFR was significantly increased compared with the db/db + Esax group (Fig. 3a). This result suggests that the corrective effect of esaxerenone on glomerular hyperfiltration was attenuated by the adenosine receptor antagonist. Furthermore, the db/db group exhibited significant afferent arteriolar dilation compared with the db/m group. In the db/db + Esax group, the afferent arteriolar dilation was significantly corrected compared with the db/db group. The significant afferent arteriolar dilation was observed in the db/db + Esax + A1aR-a group compared with the db/m + and db/db + Esax group. No significant change was detected in the diameter of the efferent arteriole in each group (Fig. 3b–d). The AA:EA ratio was elevated exclusively in the db/db and db/db + Esax group (Fig. 3e). These findings suggest that MR activation plays a role in the glomerular hyperfiltration observed in DKD and that this effect is mediated through the TGF pathway, as evidenced by the attenuation of esaxerenone’s effect following adenosine signal inhibition.

### MR activation modulates NO and adenosine signalling in MD cells

**Figure 4:**
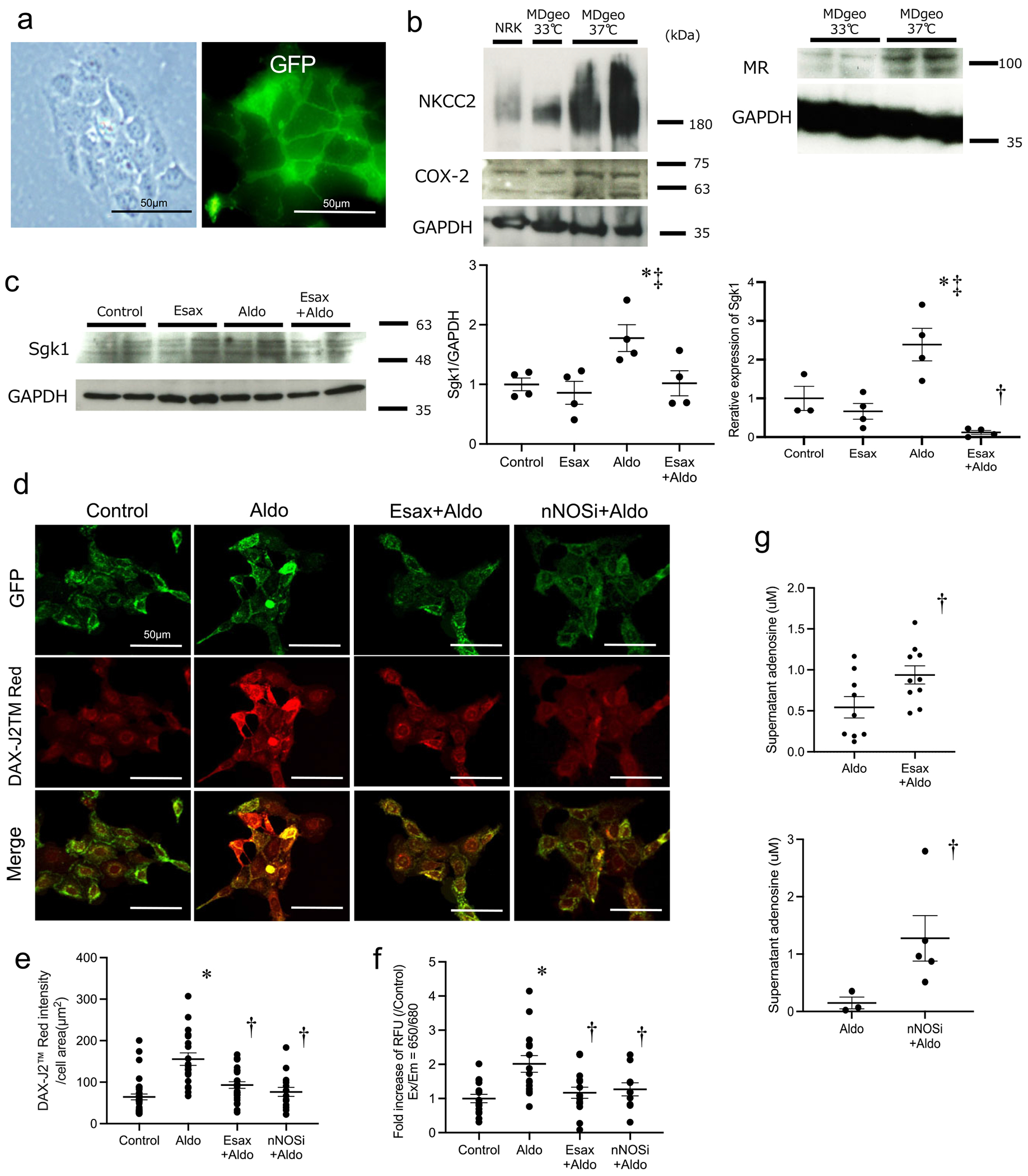
Characterization of MDgeo cells and evaluation of NO production induced by MR activation. **(a)** Stereomicroscopy and fluorescence microscopy images of differentiated MDgeo cells expressing membrane-targeted GFP. Scale bar = 50 μm. **(b)** Western blot analysis showing the protein expression of NKCC2 and COX-2 in NRK cells as well as in undifferentiated and differentiated MDgeo cells. MR expression was also examined under both conditions. **(c)** SGK1 protein expression was analysed by western blot (left) and SGK1 mRNA levels were assessed by quantitative real-time PCR (right) in MDgeo cells under control conditions or after treatment with esaxerenone (Esax), aldosterone (Aldo) or a combination of Aldo and Esax (Esax + Aldo). GAPDH was used as the loading control for western blot. Control: *n* = 3–4; other groups: *n* = 4 samples per group. **(d)** Representative fluorescence images of NO production in MDgeo cells under control, Aldo, Esax or nNOS inhibitor treatment. GFP (green) indicates the cell membrane; DAX-J2 Red (red) indicates NO. Scale bar = 50 *μ*m. **(e)** Quantification of DAX-J2 Red fluorescence intensity normalized to the cell area. *n* = 20–35 cells per group. **(f)** NO production measured using the Cell Meter Fluorimetric Intracellular NO Assay Kit. *n* = 10–15 samples per group. **(g)** Adenosine concentrations in the culture supernatant under Aldo stimulation, with either Esax or nNOS inhibition. *n* = 3–10 samples per group. Data are presented as mean ± SEM. * *P* < .05 versus control; ^†^ *P* < .05 versus Aldo; ^‡^ *P* < .05 versus Esax.

We investigated the impact of MR activation on NO production and adenosine secretion in MD cells using differentiated MDgeo cells. Differentiated MDgeo cells exhibited spindle-shaped morphology and expressed green fluorescent protein (GFP) localized to the cell membrane (Fig. 4a). Compared with undifferentiated cells, differentiated cells showed higher protein expression of NKCC2, COX-2 and MR, as confirmed by western blotting (Fig. 4b). To assess MR activation, we measured SGK1 expression by both western blotting and quantitative real-time polymerase chain reaction. SGK1 expression was significantly increased after aldosterone stimulation while pretreatment with esaxerenone attenuated this response (Fig. 4c). NO production was evaluated by two independent methods. First, we performed live-cell fluorescence imaging using an NO-sensitive dye (DAX-J2 Red), with signal intensity normalized to the cell area (Fig. 4d, e). Second, intracellular NO levels were quantified using the Cell Meter Fluorometric Assay (Fig. 4f). Aldosterone stimulation significantly increased NO production, which was reduced by both esaxerenone and an nNOS inhibitor. We also measured adenosine concentrations in the culture supernatant, a key mediator of TGF. Adenosine levels were significantly higher in esaxerenone- and nNOS inhibitor–treated groups compared with the aldosterone-only group (Fig. 4g). These results suggest that aldosterone-induced MR activation in MD cells enhances NO production and suppresses adenosine release, whereas MR blockade reverses this effect, thereby potentially restoring TGF signalling.

### MR activation decreases NKCC2 expression through nNOS-mediated NO production in MD cells

**Figure 5:**
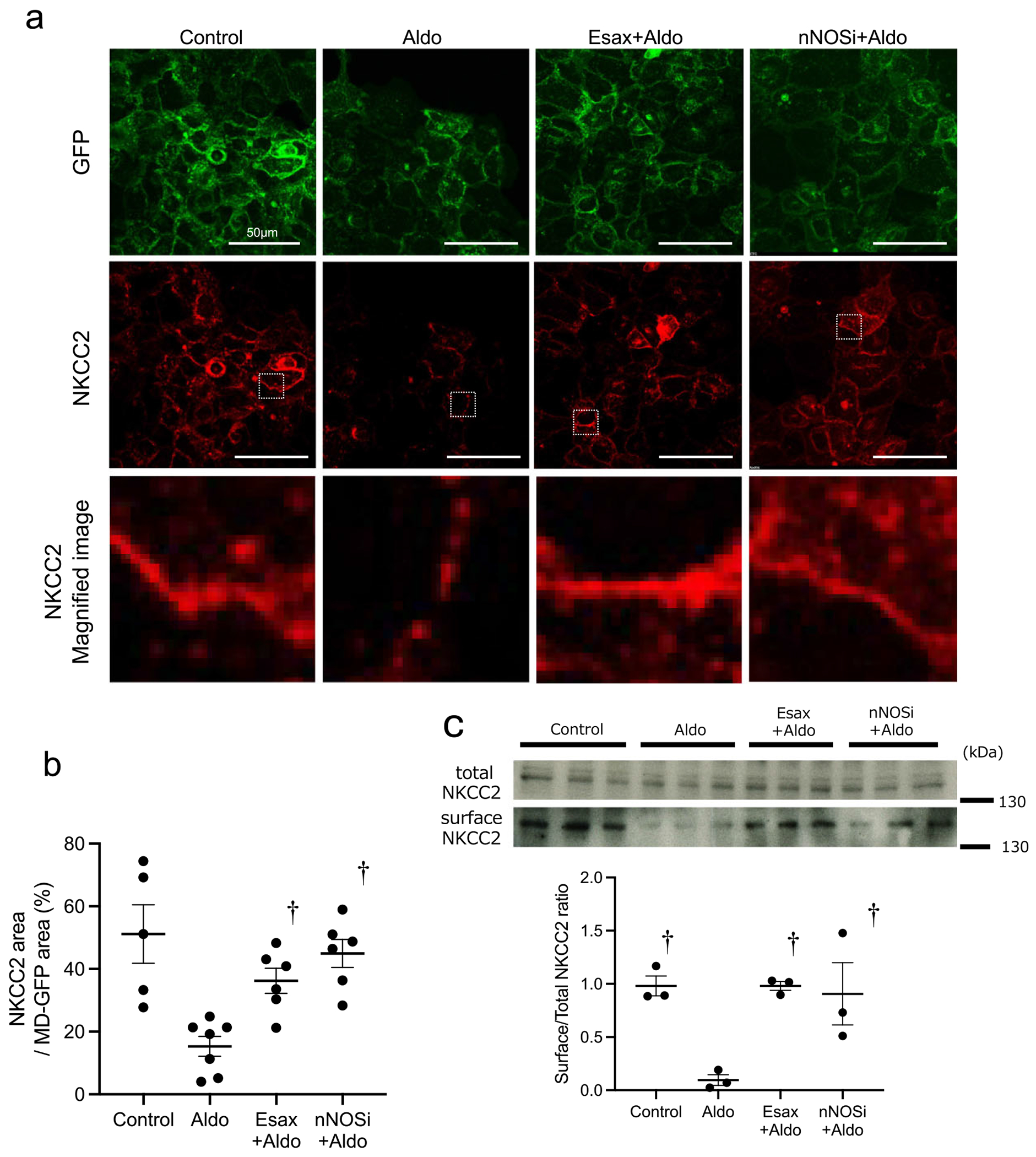
Effects of esaxerenone and an nNOS inhibitor on NKCC2 membrane expression induced by MR activation. **(a)** Representative immunofluorescence images showing NKCC2 (Alexa594, red) and the cell membrane marker GFP (green) in MD cells under control conditions, aldosterone (Aldo) treatment, Aldo with esaxerenone (Esax + Aldo) or Aldo with an nNOS inhibitor (nNOSi + Aldo). Lower panels in each group show higher magnification of NKCC2 signals. Scale bar = 50 *μ*m. **(b)** Quantification of the NKCC2 fluorescence area within GFP-positive regions by densitometry. *n* = 5–6 samples per group. **(c)** Representative immunoblots and summary bar graphs showing surface and total NKCC2 expression in the apical membrane fraction of MD cells from each treatment group. Surface NKCC2 abundance was quantified by densitometry and expressed as the ratio of surface to total NKCC2. *n* = 3 samples per group. Data are presented as mean ± SEM. ^†^ *P* < .05 versus Aldo.

The membrane expression level of NKCC2 depends on the balance between its production, trafficking and degradation. Typically, only 3–5% of NKCC2 is present on the apical membrane of thick ascending limb cells, with the majority localized in subapical compartments. The half-life of NKCC2 on the cell membrane is ≈1 hour and its levels are tightly regulated by exocytosis, endocytosis and recycling. We examined the impact of increased NO production induced by MR activation in MD cells on NKCC2 membrane expression. Immunofluorescence analysis confirmed that NKCC2 is predominantly localized on the cell membrane and its expression was reduced upon aldosterone stimulation. The magnified image revealed that NKCC2 expression became discontinuous following aldosterone treatment. However, its expression was restored following treatment with esaxerenone and an nNOS inhibitor (Fig. 5a, b). Membrane proteins were extracted from MDgeo cells and NKCC2 expression was evaluated by western blotting. Aldosterone stimulation reduced NKCC2 expression, whereas treatment with esaxerenone or an nNOS inhibitor preserved its expression (Fig. 5c). In addition, a representative western blot of glyceraldehyde 3-phosphate dehydrogenase (GAPDH) in intracellular and plasma membrane fractions is shown (see Supplementary Fig. S3), confirming the absence of GAPDH in the membrane fraction. Furthermore, additional experiments using the steroidal MRB spironolactone showed no significant differences in NO production or NKCC2 membrane localization compared with the non-steroidal MRB esaxerenone (see Supplementary Figs. S4 and S5).

### Esaxerenone maintains NKCC2 expression in MD cells of db/db mice

**Figure 6:**
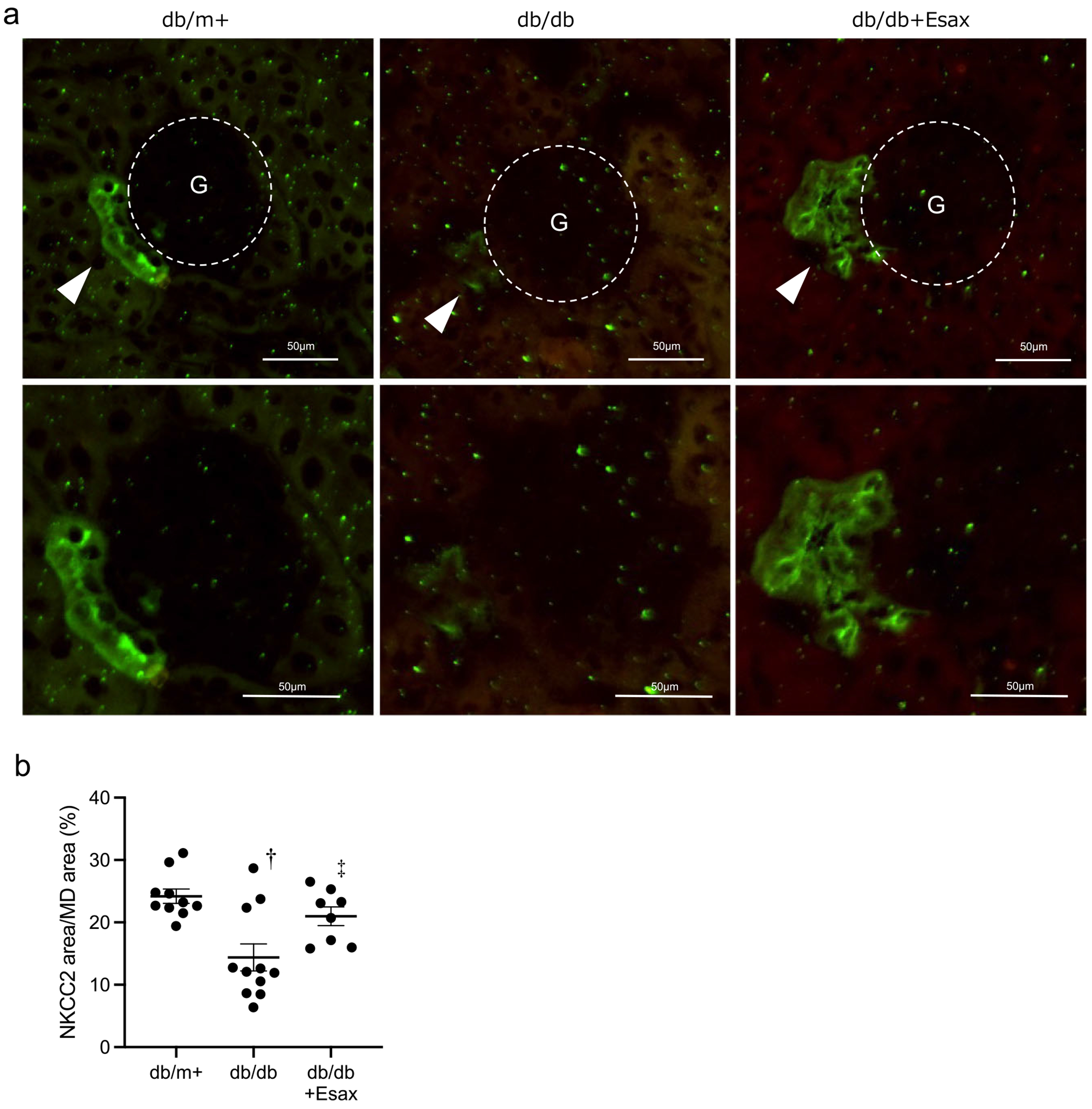
Expression of NKCC2 in MD cells in mice. **(a)** Representative images of NKCC2 immunofluorescence in kidney sections from db/m^+^, db/db and db/db + esaxerenone (Esax) mice. Glomeruli (G) are outlined with dashed circles. Arrowheads indicate proximal tubules in contact with the glomerular tuft, where MD cells reside. Scale bar = 50 *μ*m. **(b)** Quantification of the NKCC2 fluorescence area (within GFP-positive regions) normalized to the area of MD cells by densitometry. *n* = 8–11 glomeruli per group. Data are presented as mean ± SEM. ^†^ *P* < .05 versus db/m^+^; ^‡^ *P* < 0.05 versus db/db.

To evaluate NKCC2 expression in MD cells *in vivo*, we performed immunofluorescence staining on frozen kidney sections from db/m^+^, db/db and db/db mice treated with esaxerenone. In db/m^+^ mice, NKCC2 expression was clearly observed in tubular segments corresponding to the MD region. In contrast, db/db mice showed a marked reduction in NKCC2 expression in these areas. Treatment with esaxerenone preserved NKCC2 expression in the MD (Fig. 6a, b). Furthermore, recent reports have indicated that MD cells express CCN1 (CYR61), which may contribute to extracellular matrix remodelling and angiogenesis. In particular, decreased CCN1 expression has been reported in the kidneys of patients with CKD [9]. To explore the involvement of CCN1, we performed immunofluorescence staining for CCN1. Strong CCN1 signals were observed around the MD in db/m^+^ mice, whereas no detectable staining was observed in db/db or db/db mice treated with esaxerenone (see Supplementary Fig. S6). These findings suggest that the antiproteinuric effect of esaxerenone is independent of CCN1 signalling.

## DISCUSSION

The objective of this study was to elucidate the molecular mechanisms of renal protection by nsMRA in DKD. We hypothesized that MR activation in MD cells regulates the glomerular haemodynamics in DKD via a TGF mechanism and evaluated the effect of nsMRA on suppressing glomerular hyperfiltration in DKD. Systemic administration of aldosterone-induced MR activation led to the dilation of glomerular afferent arterioles in rats, promoting glomerular hyperfiltration. Esaxerenone improved glomerular hyperfiltration and reduced albuminuria in diabetic mice. This reaction was found to involve the correction of excessive dilation of glomerular afferent arterioles mediated by adenosine, a key TGF signal. Furthermore, experiments using MD cells revealed that MR activation enhanced NO production and reduced NKCC2 expression on the cell membrane. These phenomena were attenuated by esaxerenone and nNOS inhibitors. This study demonstrated that the nsMRA esaxerenone suppresses glomerular hyperfiltration in DKD by improving TGF signalling. It was also revealed that MR activation in MD cells is involved in the abnormalities of renal microcirculatory dynamics in DKD.

MR activation is known to contribute to the progression of DKD. Even when plasma aldosterone levels are low or within the normal range, MR can still be activated and promote renal injury, highlighting the therapeutic potential of MR antagonists [13]. In the present study, we focused primarily on MR signalling in the MD and therefore fed all experimental groups (db/m^+^, db/db and db/db mice treated with esaxerenone) the same high-salt diet to uniformly enhance MR activation. Fujita *et al.* [14] reported that high-salt loading caused disproportionate elevations in plasma aldosterone levels in obese spontaneously hypertensive rats compared with lean rats. Furthermore, in Dahl salt-sensitive rats, a high-salt diet induced hypertension and proteinuric renal injury despite a reduction in plasma aldosterone, accompanied by increased renal MR signalling including upregulation of SGK1 and epithelial sodium channel [15]. These findings indicate that in the context of obesity or diabetes, high salt intake can activate MR through both aldosterone-dependent and Rac1-mediated pathways [12]. Consistent with these reports, our preliminary experiments under a normal-salt diet (see Supplementary Fig. S7) showed only mild mesangial expansion and glomerular hyperfiltration in db/db mice, with no significant effect of esaxerenone, supporting the importance of high salt loading for unmasking MR-dependent changes in TGF and glomerular haemodynamics in this model.

MR is widely expressed in various renal cell types, including podocytes, mesangial cells, tubular epithelial cells and proximal tubular cells [5]. Although our study primarily addressed MR signalling in the MD, MR activation in vascular smooth muscle cells may also contribute to diabetic hyperfiltration independent of A1aR signalling. A previous study using micropuncture in rabbits demonstrated that aldosterone infusion induced concentration-dependent MR activation, causing constriction of both afferent and efferent arterioles, with stronger contraction in the efferent arteriole [16]. This mechanism could theoretically promote glomerular hyperfiltration. However, in both the aldosterone-infusion and diabetic models used in the present study, we consistently observed afferent arteriolar dilation. This discrepancy may be explained by the experimental conditions: in the aforementioned rabbit study, constriction appeared within 5 minutes of aldosterone administration, indicating a rapid, non-genomic vasoconstrictive effect, whereas our experiments addressed chronic MR activation. Under our conditions, regulation by TGF appears to predominate. To our knowledge, no previous study has directly demonstrated the involvement of MR activation in glomerular hyperfiltration associated with DKD. Our findings show for the first time that MR antagonism corrects afferent arteriolar overdilation and suppresses hyperfiltration. Moreover, the observed changes in urinary adenosine with nsMRA treatment and the effects of an A1aR antagonist strongly suggest that MR activation modulates TGF signalling.

MDgeo cells closely resemble actual MD cells and are an optimal *in vitro* model for studying TGF signalling. In this study, MR activation in MDgeo cells increased NO production and decreased adenosine secretion into the supernatant. Additionally, reduced NKCC2 membrane expression was observed. Previous studies have suggested that NO suppresses NKCC2 membrane trafficking via the cGMP-PDE2 pathway and promotes its degradation through the ubiquitin-proteasomal system [17–19]. This cGMP-PDE2 pathway is also activated by natriuretic peptides and ET-1. Furthermore, angiotensin II has been reported to inhibit NO production via ET-1, reduce NO-dependent NKCC2 suppression and promote NKCC2 phosphorylation, thereby enhancing its activity [20]. While this study did not elucidate the detailed molecular mechanisms of NKCC2 activity regulation, these previous reports support our findings and provide a plausible mechanistic explanation. Aldosterone treatment in MDgeo cells consistently increased NO production. Since MR activation did not alter nNOS expression levels (data not shown), it is expected that nNOS activity was enhanced. NKCC2 activity is regulated by its membrane expression and phosphorylation. The findings suggest that MR activation in MD cells influences NKCC2 membrane expression through increased NO production.

### Limitations

This study has several limitations. First, aldosterone was used to investigate the effects of MR activation on renal microcirculatory dynamics. However, aldosterone is known to have non-genomic pathways independent of MR, making it difficult to distinguish between the effects of MR activation itself and the specific actions of aldosterone. Therefore, further experiments using other MR ligands, such as glucocorticoids or Rac1-mediated MR activation, are needed. Second, while this study demonstrated that MR activation in MD cells contributes to glomerular hyperfiltration, it remains unclear whether MR activation in MD cells alone is sufficient to induce hyperfiltration. The kidney contains many MR-expressing cells, such as podocytes, mesangial cells and proximal tubular cells, that may also contribute to the observed effects. Therefore, evaluating the impact of MR activation in non-MD cells is a crucial future direction. Third, in the imaging analysis conducted in this study, we selected the most intact nephrons for analysis. However, it is possible that the physiological conditions of the nephrons were not entirely preserved. Changes in haemodynamics and tissue stress responses under experimental conditions could have influenced the data. Future studies should incorporate *in vivo* models to validate the findings under more physiological conditions. These limitations should be considered when interpreting our findings. Finally, all experiments were performed exclusively in male mice, and the potential influence of sex differences cannot be excluded.

## CONCLUSION

We investigated the renoprotective mechanisms of nsMRA in DKD and demonstrated that MR activation contributes to glomerular hyperfiltration. Specifically, we demonstrated that MR activation regulates glomerular microcirculatory dynamics via TGF in MD cells.

## Supplementary Material

Supplemental Material

Supplementary data are available at Nephrology Dialysis Transplantation online.

## Figures and Tables

**Table 1:** Physical data.

Variable	Control	Aldo	Aldo + Esax
sBP (mmHg)	96.9 ± 1.8	115.8 ± 1.4^†^	101.7 ± 2.2^‡^
HR (bpm)	425 ± 40	371 ± 28	360 ± 1
BW (g)	287 ± 7	344 ± 15	293 ± 4

Values are expressed as mean ± SEM. n = 4–6 per group.

Aldo: aldosterone; Esax: esaxerenone; sBP: systolic blood pressure; bpm: beats per minute; BW: body weight.

† *P* < .05 versus control;

‡ *P* < .05 versus Aldo.

**Table 2:** Physical data and biochemical data.

Variable	db/m^+^	db/db	db/db + Esax
sBP (mmHg)	121 ± 2	97 ± 6^†^	98 ± 5^†^
HR (bpm)	564 ± 22	362 ± 21^†^	449 ± 19^†^
BW (g)	32.8 ± 0.3	45.7 ± 1.6^†^	42.5 ± 1.2^†^
SGlu (mg/dl)	173 ± 5	674 ± 40^†^	702 ± 31^†^
SK (mEq/l)	5.5 ± 0.1	5.3 ± 0.4	5.4 ± 0.2
SCre (mg/dl)	0.10 ± 0.01	0.08 ± 0.01^†^	0.08 ± 0.01^†^

Values are expressed as mean ± SEM. *n* = 6–8 per group.

Esax: esaxerenone; sBP: systolic blood pressure; bpm: beats per minute; BW: body weight; SGlu: serum glucose; SK: serum potassium; SCre: serum creatinine.

† *P* < .05 versus db/m^+^.

## Data Availability

All animal experiments were conducted at Kawasaki Medical School. All data supporting the findings of this study are available in the main text and supplementary information. Additional information is available from the corresponding author upon reasonable request.

## KEY LEARNING POINTS

### What was known:

- Mineralocorticoid receptor antagonists are known to reduce albuminuria in diabetic kidney disease (DKD), but the precise mechanisms underlying their renoprotective effects, particularly related to glomerular haemodynamics and tubuloglomerular feedback (TGF), have not been fully clarified.

### This study adds

- This study demonstrates that esaxerenone restores TGF function and attenuates glomerular hyperfiltration by modulating nitric oxide and adenosine signalling, leading to reduced albuminuria in DKD. These effects were confirmed using *in vivo* multiphoton imaging and cellular experiments.

### Potential impact:

- These findings highlight the therapeutic potential of esaxerenone in DKD through restoration of TGF and reduction of hyperfiltration, providing new mechanistic insights and suggesting a novel strategy for addressing residual risk in DKD beyond current therapies.
