# Supplementary material for: The non-steroidal mineralocorticoid receptor blocker esaxerenone reduces glomerular hyperfiltration and albuminuria
Source: Nephrol Dial Transplant. 2025 Nov 6;41(6):1069–81. doi: 10.1093/ndt/gfaf232 (PMC13165900; doi:10.1093/ndt/gfaf232)

**Supplementary Information**

**Effects of Nonsteroidal Mineralocorticoid Receptor Blocker Esaxerenone on Renal Glomerular Hemodynamics in Diabetic Kidney Disease**

Rie Tatsugawa, Kengo Kidokoro, Tsukasa Iwakura Akira Hirano, Eriko Kajimoto, Masanobu Takasu, Masafumi Wada, Yoshihisa Wada, Georgina Gyarmati Seiji Kishi, Hajime Nagasu, David Z. I. Cherney, Janos Peti-peterdi, Tamaki Sasaki, Naoki Kashihara

1 supplementary Method, and 7 supplementary figure.

**Supplementary Methods**
Male db/db mice (C57BLKS/J background, 10 weeks old) were maintained on a normal-salt diet and randomly assigned to vehicle (n = 10) or esaxerenone (n = 10) groups. Esaxerenone was administered orally at 3 mg/kg/day for 4 weeks; controls received vehicle.Urinary protein excretion and single-nephron GFR were measured as described in the main Methods. Kidney sections were examined by PAS staining to evaluate mesangial expansion. All procedures were approved by the Ethics Review Committee for Animal Experimentation at Kawasaki Medical School (21-088, 24-066).

**Supplementary Figure 1 | Blood pressure changes in db/m⁺, db/db, and db/db + Esax mice**
Blood pressure was measured at 8, 12, and 15 weeks of age in db/m⁺, db/db, and db/db + esaxerenone (Esax) mice. Each line represents an individual mouse (n = 6–8 per group).

**Supplementary Figure 2 | Glomerular structural changes and renal fibrosis markers in db/m⁺, db/db, and db/db + esaxerenone (Esax) mice**
(a) Mesangial matrix area relative to glomerular area. (b) Thickness of the glomerular basement membrane (GBM). (c) Glomerular cross-sectional area. (d) Representative histological images of kidney sections from the indicated groups. Upper panels: Masson’s trichrome staining (scale bar = 500 µm) showing overall renal morphology and interstitial fibrosis. Middle panels: immunohistochemical staining for α-smooth muscle actin (α-SMA; scale bar = 50 µm). Lower panels: immunohistochemical staining for transforming growth factor-β (TGF-β; scale bar = 50 µm).


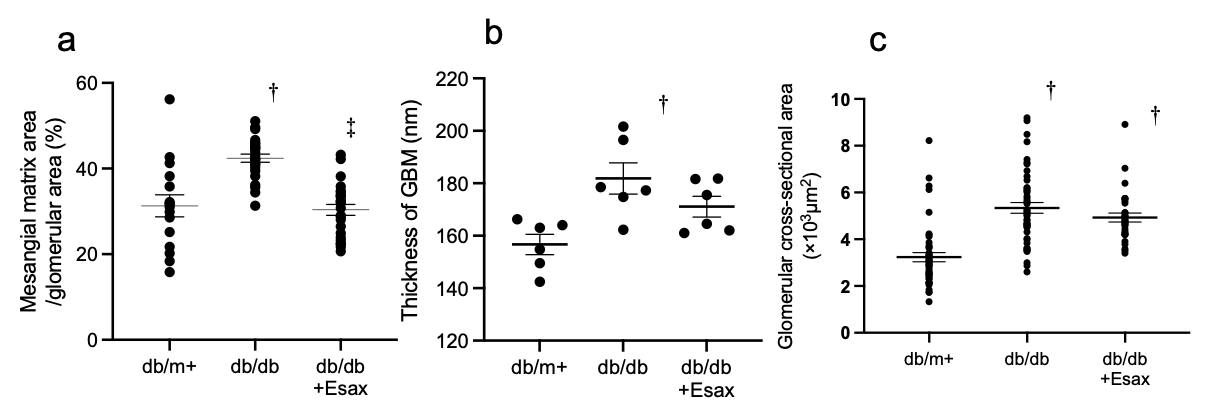

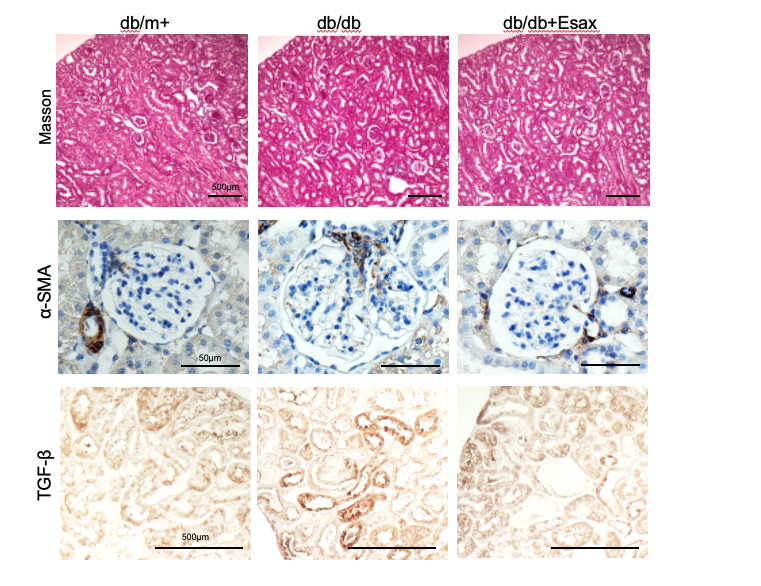


**Supplemental Figure 3 | Representative immunoblots of GAPDH in intracellular and plasma membrane fractions of MDgeo cells.**

Western blot analysis of GAPDH in intracellular (upper panel) and plasma membrane (surface) fractions (lower panel) prepared from MDgeo cells.Strong GAPDH signal was detected in the intracellular fraction, whereas little to no signal was observed in the plasma membrane fraction, confirming the absence of GAPDH in the membrane fraction.


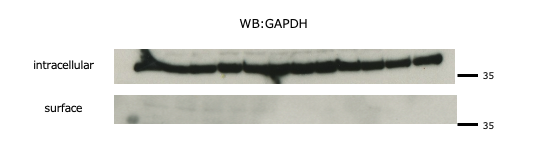


**Supplemental Figure 4 | Effects of spironolactone on Aldo–induced NO production in MDgeo cells**
Representative fluorescence images of differentiated MDgeo cells expressing plasma membrane–targeted GFP (green) and the NO indicator DAX-J^2TM Red (red) under the indicated treatment conditions (Control, Aldo, and Spironolactone + Aldo). Merged images show the overlay of GFP and DAX-J^2TM Red signals. Scale bar = 50 µm. Quantification of DAX-J^2TM Red fluorescence intensity within GFP-positive regions is shown in the lower panel. Each dot represents an individual sample; horizontal bars indicate mean ± SEM. †P < 0.05 vs. Control; ‡P < 0.05 vs. Aldo.


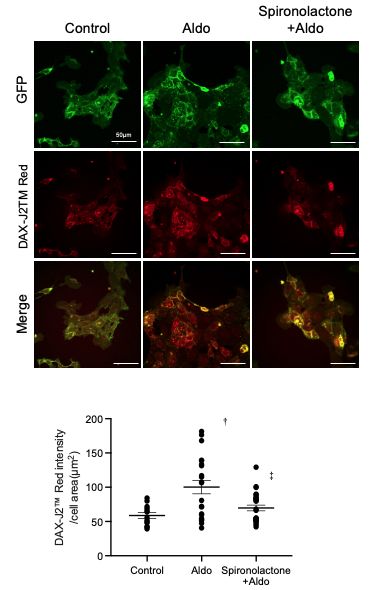


**Supplemental Figure 5 | Effects of spironolactone on Aldo–induced changes in NKCC2 membrane expression in MDgeo cells**
Representative immunofluorescence images of differentiated MDgeo cells expressing plasma membrane–targeted GFP (green) and stained for NKCC2 with Alexa594 (red) under the indicated treatment conditions (Control, Aldo, and Spironolactone + Aldo). Scale bar = 50 µm. Quantification of NKCC2-positive area within GFP-positive regions is shown in the lower panel. Each dot represents an individual sample; horizontal bars indicate mean ± SEM. †P < 0.05 vs. Control.


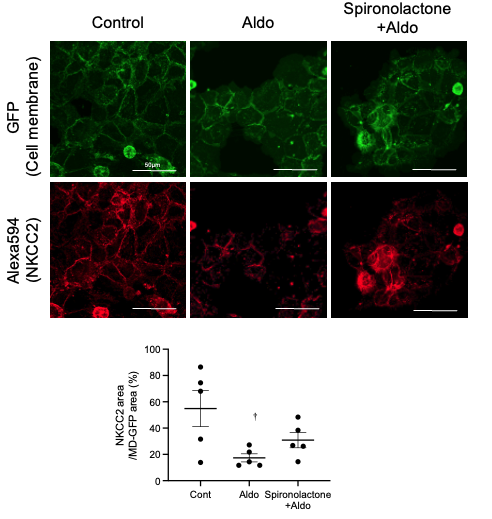


**Supplemental Figure 6| Immunofluorescence staining of CCN1 in kidney sections from db/m⁺, db/db, and db/db + Esax mice**
Representative images of CCN1 immunofluorescence (red) in kidney sections. Nuclei were counterstained with DAPI (blue). Dashed circles indicate glomeruli (G), and arrowheads denote CCN1-positive signals in the macula densa region. Scale bar = 100 µm.


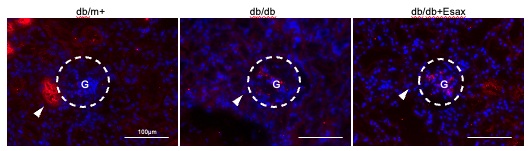


**Supplementary Figure 7 | Effects of esaxerenone on glomerular morphology and function in db/db mice without high-salt loading (HS−)**
(a) Representative images of periodic acid–Schiff (PAS) staining (left panels; scale bar = 50 µm) and ultrastructural changes observed by electron microscopy (right panels; scale bar = 1.0 µm) in db/db and db/db + esaxerenone (Esax) mice without high-salt (HS) loading. (b) Mesangial matrix area relative to glomerular area. (c) Urinary albumin excretion. (d) Single-nephron glomerular filtration rate (SNGFR).
Each dot represents an individual mouse; horizontal bars indicate mean ± SEM. ‡P < 0.05 vs. db/db.


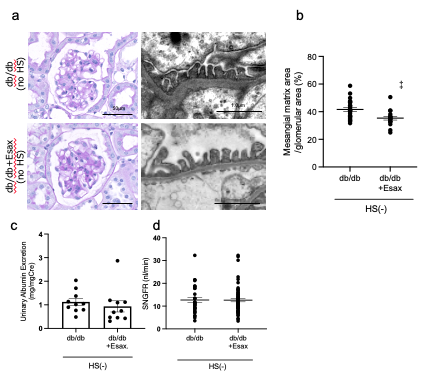

Supplement: gfaf232_Supplemental_File [file gfaf232_Supplemental_File.docx]
